# Cardiac Safety of Ozanimod Use, a Novel Sphingosine-1-Phosphate Receptor Ligand, in COVID-19 Patients Requiring Oxygen: Secondary Analysis of the COZI Randomized Clinical Trial

**DOI:** 10.1016/j.cjco.2024.05.002

**Published:** 2024-05-16

**Authors:** Guillaume Domain, Pascale Blais-Lecours, Camille Strubé, Nicolas Dognin, Nathalie Châteauvert, Noémie Savard, Tuyen Nguyen, Philippe Rola, David Marsolais, François Lellouche, Jean-François Sarrazin

**Affiliations:** a Institut universitaire de cardiologie et de pneumologie de Québec— Université Laval, Québec, Québec, Canada; b Centre de recherche de l’Institut universitaire de cardiologie et de pneumologie de Québec—Université Laval, Québec, Québec, Canada; c Cite-de-la-Santé Hospital, CISSS de Laval, Laval, Québec, Canada; d Santa Cabrini Hospital, CIUSSS EMTL, Montréal, Québec, Canada

## Abstract

**Background:**

Ozanimod is a novel immune modulator that could be useful in viral pulmonary infections by reducing lung inflammation. It is an S1P receptor ligand known to induce bradycardia and more serious adverse cardiac effects, such as atrioventricular block and QT interval prolongation. We present a substudy of the **C**OVID-19 **Oz**animod **I**ntervention (COZI) trial in which ozanimod was administered in acute pulmonary infection patients, to assess cardiac safety.

**Methods:**

In this pilot randomized open-label trial, COVID-19 patients requiring oxygen support were randomized into 2 groups: standard-of-care + ozanimod (OZA) vs standard-of-care alone (SOC). All patients were monitored with a 14-day electrocardiogram monitor (CardioSTAT, Icentia, Quebec, QC) during their hospitalization. We evaluated the cardiac effects of ozanimod on heart rate (HR), PR interval length, and QT interval duration.

**Results:**

A total of 42 patients were analyzed: 23 in the SOC group and 19 in the OZA group. Mean hourly HR over the first 10 days of treatment decreased in the OZA group, compared with that in the SOC group (*P* < 0.0001). The maximum decrease in HR occurred on day 3. The maximum decrease in HR occurred on day 3, without a significant difference between groups: 49 beats per minute (interquartile range, 42-59) in the OZA group, and 54 beats per minute (48–60) in the SOC group, *P* = 0.45. No high-degree atrioventricular block was recorded. QT and PR interval median values were within the normal range in both groups, without a significant difference.

**Conclusions:**

The maximal reduction in HR occurred 3 days after the onset of ozanimod treatment in patients hospitalized for COVID-19, but it did not remain significant over the 10-day treatment period. No relevant cardiac adverse event was observed.

## Résumé

**Contexte:**

L’ozanimod est un immunomodulateur novateur qui pourrait être utile dans le traitement des infections pulmonaires d’origine virale en réduisant l’inflammation pulmonaire. Il s’agit d’un ligand des récepteurs de la sphingosine-1-phosphate (S1P) connu pour induire une bradycardie et avoir des effets cardiaques indésirables plus graves, comme le bloc auriculoventriculaire et l’allongement de l’intervalle QT. Nous présentons une sous-étude de l’essai COZI (COVID-19 Ozanimod Intervention) dans le cadre de laquelle l’ozanimod a été administré à des patients atteints d’une infection pulmonaire aiguë pour évaluer l’innocuité cardiaque.

**Méthodologie:**

Dans le cadre de cette étude pilote ouverte à répartition aléatoire, des patients atteints de la COVID-19 nécessitant une oxygénothérapie de soutien ont été répartis en deux groupes : soins standard + ozanimod (OZA) et soins standard uniquement. Tous les patients ont été surveillés en continu pendant 14 jours à l’aide d’un moniteur ECG (CardioSTAT, Icentia, Québec [Québec]) durant leur hospitalisation. Nous avons évalué les effets de l’ozanimod sur la fréquence cardiaque, la longueur de l’intervalle PR et la durée de l’intervalle QT.

**Résultats:**

Au total, 42 patients ont été analysés : 23 dans le groupe ayant reçu les soins standard et 19 dans le groupe OZA. La fréquence cardiaque horaire moyenne au cours des 10 premiers jours de traitement a diminué dans le groupe OZA, comparativement au groupe ayant reçu les soins standard uniquement (*p* < 0,0001). La diminution maximale de la fréquence cardiaque a été observée le jour 3, sans différence significative entre les groupes : 49 battements par minute (plage interquartile : 42 à 59) dans le groupe OZA et 54 battements par minute (48 à 60) dans le groupe ayant reçu les soins standard uniquement (*p* = 0,45). Aucun bloc auriculoventriculaire de haut degré n’a été enregistré. Les valeurs médianes des intervalles QT et PR se situaient dans la plage normale dans les deux groupes, sans différence significative.

**Conclusions:**

La réduction maximale de la fréquence cardiaque a été observée 3 jours après l’instauration du traitement par l’ozanimod chez les patients hospitalisés pour la COVID-19, mais n’est pas demeurée significative durant la période de traitement de 10 jours. Aucune manifestation cardiaque indésirable pertinente n’a été observée.

Ozanimod is a novel oral sphingosine-1-phosphate (S1P) receptor ligand (SRL) that binds selectively to the S1P 1 and 5 receptors (S1P_1_, S1P_5)._^1^, ^2^, ^3^ SRLs, including S1P_1_-specific ligands, have been determined to be able to reduce lung inflammation in various lung-disease models.^4^, ^5^, ^6^

S1P receptors are also instrumental to the regulation of microvasculature permeability, vascular tone, heart rate (HR), and cardiac repolarization.^7^ Several SRLs and SRL prodrugs were associated with bradycardia and more-severe adverse cardiac events, such as atrioventricular block (AVB) and QT prolongation. In the ozanimod first-in-human, single-ascending-dose and multiple-ascending-dose studies, a transient, dose-dependent decrease in HR was observed.^8^

The prospective randomized open-label **C**OVID-19 **Oz**animod **I**ntervention (COZI) pilot trial was conducted in 3 Canadian hospitals. Patients admitted for COVID-19 requiring oxygen were eligible. The primary endpoint was an investigation for size effect, and variance over time was the assessment of safety and efficacy. This study shows for the first time that this new pharmacologic agent may be safely administered to patients hospitalized for viral pneumonia, with potential clinical benefits.^9^

The objectives of this cardiac substudy of the COZI trial were to evaluate the impact of ozanimod on HR variations and various electrocardiogram (ECG) parameters, especially the QT interval and the PR duration.

## Methods

An open-label, prospective, randomized pilot clinical trial (ClinicalTrials.gov #NCT04405102) was carried out in 3 Quebec (Canada) centres. The study was approved by an institutional review board (REB# MP-10-2021-3474) and was conducted in accordance with the principles set forth in the Declaration of Helsinki. All subjects provided written informed consent before participating in the study. The study was conducted between September 2020 and May 2022.

Patients were randomized into 2 groups: standard-of-care + ozanimod (the OZA group) vs standard-of-care alone (the SOC group). Patients assigned to the OZA group received ozanimod 0.23 mg on days 1-4, and ozanimod 0.46 mg daily for a maximum of 10 days, in addition to the standard-of-care. The control SOC group was managed with the standard-of-care alone. Randomization was stratified based on risk factors of poor outcomes and on the utilization or not of high-flow nasal canula at inclusion (age > 65 years, male gender, severe cardiac disorders, known chronic diseases). To evaluate cardiac safety, during hospitalization, all patients were monitored with a 14-day single-lead ECG monitor, the CardioSTAT (Icentia, Quebec, QC), and telemetry for the first 4 days after inclusion. Electrolyte anomalies were monitored and corrected when necessary.

### Inclusion criteria

Patients with hypoxemia related to SARS-CoV-2 viral pneumonia requiring oxygen and satisfying the following criteria were included: having a confirmed COVID-19 (a polymerase chain reaction test positive for COVID-19); being aged > 18 years but < 85 years; having a body mass index > 20 but < 40; having hypoxemia related to viral pneumonia (COVID-19) requiring oxygen or nasal high-flow therapy (to maintain oxygen saturation > 92%), without criteria for immediate intubation or need for other respiratory supports; having initiation of oxygen supplementation < 72 hours; having a creatinine clearance (Chronic Kidney Disease Epidemiology Collaboration [CKD EPI]) of > 30 mL/min per 1.73 m^2^; having a serum troponin i < 80 ng/L; and having a HR ≥ 55 beats per minute (bpm) if beta-blockers or calcium-channel blocker nondihydropyridine are used, and ≥ 60 bpm in the other patients.

### Exclusion criteria

Patients who had the following were excluded: admission for palliative care; agitation; severe untreated sleep apnea; history of or currently active primary or secondary immunodeficiency; recent (within the preceding 6 months) occurrence of myocardial infarction; unstable angina; stroke; transient ischemic attack; decompensated heart failure requiring hospitalization; class III or IV heart failure; known presence of Mobitz type II second-degree or third-degree AVB; sick sinus syndrome, or sinoatrial block, unless patient has a functioning pacemaker; cirrhosis Child-Pugh score class C; pregnancy; lactation; or persistent hypotension. Patients requiring treatment with class Ia and III antiarrhythmics or concomitant use of a monoamine oxidase inhibitor also were excluded.

### Management of drugs with potential cardiac effect

Several drugs were contraindicated in the study. Due to the potential additive effects on HR and the QT interval, use of class Ia and class III antiarrhythmic drugs was prohibited during the treatment with ozanimod. Due to the potential impact on the QT interval, chloroquine, hydroxychloroquine, and azithromycin were not used in the study. In addition, concomitant use of ozanimod with strong cytochrome P450 2C8 (CYP2C8) inhibitors or inductors, such as gemfibrozil and rifampicin, respectively, were not recommended. Coadministration of monoamine oxidase inhibitors (eg, selegiline, phenelzine, tranylcypromine) with ozanimod was not recommended. When patients were treated with beta-blockers at admission, the recommendation was to reduce the dosage for patients randomized into the OZA group.

### Outcomes

We evaluated the cardiac effects of ozanimod from CardioSTAT monitoring. The values of HR, PR length, and QT interval duration of each patient were analyzed for the duration of the hospitalization or up until day 10; a high level of missing data occurred after that time point. Baseline was defined as the mean HR of the first hour recorded with the CardioSTAT device. We applied the commonly used Bazett HR correction formula. Given that our work is focused on the study of bradycardia, the overcorrection for high rates was not a problem.^10^ We registered all episodes of atrial fibrillation (AF), ventricular tachycardia, conduction disorders, and pauses.

### Statistical analysis

Baseline characteristics are presented as continuous variables (mean ± standard deviation), or as median with its associated interquartile range (IQR), according to the variable distributions. Categorical variables are presented as absolute and relative frequencies.

HR data were recorded every hour from day 1 at 10 AM (day of admission) to day 10 at 12 PM, or until discharge if this occurred before day 10. The graphical representation of HR per hour recorded daily suggested a linear mixed model with 3 fixed factors to compare the 2 groups (first fixed factor). A second fixed factor was associated to the 24 measurements obtained every hour during a day. The third fixed factor was the comparison among the 10th days. A random intercept was added to the statistical model. The dependence between repeated measurements was modeled using an autoregressive covariance matrix of correlation. Several statistical models with different interaction terms were investigated. Based on the Akaike information criterion, only one interaction term between the group effect and the time effect was added to the model. The fixed-factor days had no interaction term with the other 2 fixed factors. The PR and QTc interval data were measured 3 times a day—at 8 AM, 12 AM, and 8 PM—from day 1 to day 10 after admission, or until discharge if this occurred before day 10. Each measurement was done at equally spaced times (hours). The statistical approach suggested a hierarchical model with correlating measurements on 2 levels—days within subjects and hours within days. Three different random effects were defined, allowing covariances to vary due to the subject, days, and hours, and resulting in covariances among each of 30 measurements for a given subject. To compare the SOC group with the OZA group, a fixed effect was added to the statistical model. Two-by-two interaction terms were added among group day and hours effects. Statistical significance was considered to be a 2-tailed *P* < 0.05. Analyses were performed using SAS version 9.4 (SAS Institute, Cary, NC).

## Results

A total of 43 patients were enrolled in the COZI study, as follows: 23 in the control SOC group, and 20 in the OZA group. In one patient in the OZA group, the CardioSTAT device was lost, and the patient was excluded from the cardiac analysis. Of the 19 patients analyzed in the OZA group, 13 were hospitalized long enough to receive the dose increase on day 5. In one patient, the dose increase was delayed to day 6. The median total treatment duration is 7.5 days (range: 4.75-9). Two patients were transferred to another centre, and the treatment was interrupted at day 5 and day 13. One patient had asymptomatic second-degree type 1 AVB on day 7; thus, the dosage was down-titrated to 0.23 mg daily until hospital discharge on day 9. For another patient, ozanimod use was discontinued on day 2, due to nocturnal pause.

### Baseline characteristics

No striking difference was noted between groups regarding baseline characteristics. The median (IQR 25-75) age was 62 years (50-69) in the SOC group, and 65 years (58-68) in the OZA group. The median HR, the PR duration or QT duration at baseline, recorded at the CardioSTAT installation, are detailed in Table 1.

**Table 1:** Baseline characteristics (randomized sample)

Characteristic	SOC (n = 23)	Ozanimod (n = 19)
Age, y	59 ± 12	63 ± 11
Male	17 (74)	11 (58)
High blood pressure	8 (35)	6 (32)
Dyslipidemia	3 (13)	5 (26)
Diabetes	3 (13)	7 (37)
Coronary artery disease	0	1 (5)
Sleep apnea	4 (17)	4 (20)
Atrial fibrillation	0	1 (5)
Valve replacement	0	1 (5)
Bundle branch block	2 (9)	2 (11)
Bradycardia medication	3 (13)	3 (15)
Increase QT drugs	10 (43)	8 (42)
Heart rate, bpm	77 [72–87] (137)∗	77 [72–92] (101)∗
PR duration, msec	140 [123–152] (194)∗	146 [125–161] (177)∗
QT duration, msec	441 [432–454] (480)∗	446 [423–466] (478)∗

Continuous data were expressed as mean and standard deviation, or median and interquartile range [25–75]. Qualitative variables were presented as n (%).

SOC, standard-of-care.

∗ Maximum value.

### Single-lead ECG monitoring

**Figure 1:**
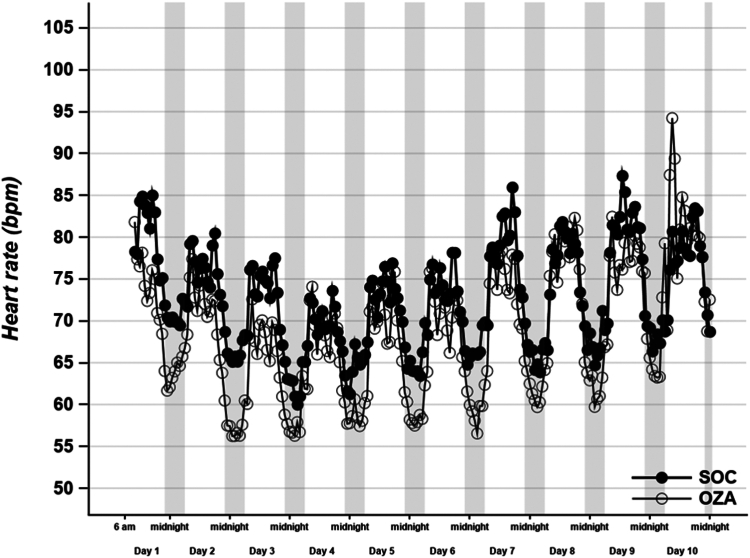
Heart rate evolution over the 10 first days of ozanimod treatment. Mean hourly data are shown for SOC (**black dots**) and OZA (**clear dots**) groups. **Grey vertical bars** show night periods (from 10 PM to 6 AM), *P* < 0.0001. bpm, beats per minute; OZA, standard-of-care + ozanimod; SOC, standard-of-care alone.

The median (IQR 25-75) monitoring duration was 194 hours 44-229) in the SOC group, and 164 hours (78-195) in the OZA group (*P* = 0.85). The 4 days of telemetry data were consistent with the CardioSTAT data. Mean hourly HR over the first 10 days of treatment is lower in the OZA group compared to that in the SOC group (*P* < 0.0001), and the maximum decrease in HR occurred on day 3 (Fig. 1).

The median HR over the first 10-day recording time had only a trend for a lower HR in the OZA group, compared to that in the SOC group (66 bpm [IQR 59-75] vs 71 bpm [IQR 68-79]; *P* = 0.10). However, the maximal HR was significantly lower (92 bpm [IQR 84-100] vs 97 bpm [IQR 85-114], respectively; *P* = 0.05). The median length of time spent with an HR < 60 bpm was significantly higher in the OZA group (44% [13%-52%]) vs the SOC group (13% [IQR 3%-33%]; *P* = 0.02). However, the minimum HR showed no significant difference between the OZA group and the SOC group (49 bpm [IQR 42-59] vs 54 bpm [IQR 48-60], respectively; *P* = 0.45; Table 2). Mean HR trended to being lower on day 3 in the OZA group (60 bpm [IQR 54-70] vs 69 bpm [IQR 63-75], respectively, *P* = 0.08).

**Table 2:** Single-lead patch monitoring results and clinical course

Measure	SOC (n = 23)	Ozanimod (n = 19)	*P*
Monitoring duration over the first 10 days, h	194 [44–229] (232)∗	164 [78–195] (228)∗	0.85
HR, bpm			
Total median	71 [68–79] (103)∗	66 [59–75] (98)∗	0.10
Minimum	54 [48–60] (74)∗	49 [42–59] (94)∗	0.45
Maximum	97 [85–114] (153)∗	92 [84–100] (130)∗	0.05
< 50 (no. of patients)	7 (30)	10 (53)	0.21
< 60 (no. of patients)	19 (83)	16 (84)	1.00
< 60, % duration	13 [3–33] (64)∗	44 [13–52] (97)∗	0.02
> 100 (no. of patients)	10 (44)	4 (21)	0.19
> 100, % duration	3 [2–11] (85)∗	8 [6–29] (50)∗	0.76
Day 3	69 [63–75] (96)∗	60 [54–70] (88)∗	0.08
Duration, msec			
PR	140 [132–155] (194)∗	146 [131–165] (193)∗	0.27
PR, day 3	141 [127–161] (209)∗	145 [131–165] (201)∗	0.68
QT	442 [435–451] (491)∗	434 [419–454] (476)∗	0.18
QT, day 3	442 [433–451] (491)∗	441 [423–457] (492)∗	0.45
Pause > 2 s	1 (4)	5 (26)	0.08
Pause > 3 s	0	1 (5)	0.46
Second-degree AV block	0	1 (5)	0.46
Nonsustained VT	2 (8)	3 (15)	0.65
Atrial fibrillation > 30 s	3 (13)	2 (10)	0.74
Need for intubation	5 (22)	1 (5)	0.10
Arterial pressure, mm Hg			
Systolic	117 [107–130]	126 [116–137]	< 0.01
Diastolic	69 [61–76]	72 [65–72]	< 0.01
Mean	85 [78–93]	91 [83–97]	< 0.01

Continuous data were expressed as median and interquartile range [25–75]. Qualitative variables were presented as n (%). no. of patients*,* number of patients with at least one episode.

AV, atrioventricular; HR, heart rate; SOC, standard-of-care; VT, ventricular tachycardia.

∗ Maximum value.

The HR drop due to ozanimod respects the HR nychthemeral variation (Fig. 1). No sustained bradycardia < 30 bpm was recorded.

**Figure 2:**
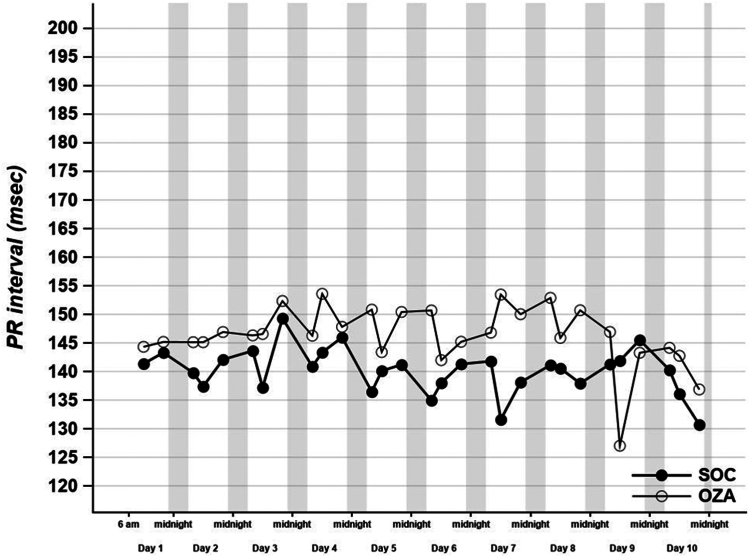
Effect of standard-of-care and ozanimod on PR interval duration (msec). PR was measured with the CardioSTAT (Icentia, Quebec, QC) device at 8:00, 12:00, and 20:00 daily. Mean ± SEM. *P* = 0.44. OZA, standard-of-care + ozanimod; SOC, standard-of-care alone.

**Figure 3:**
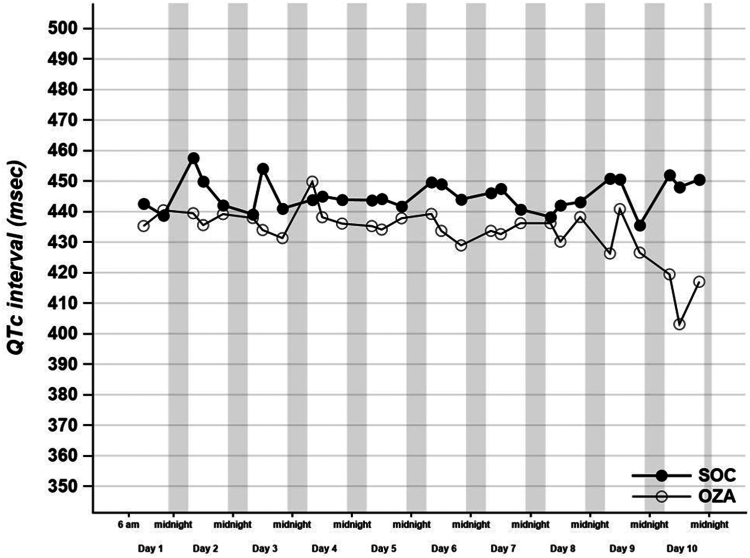
Effect of standard-of-care and ozanimod on QT interval duration (msec). QTc was measured with the CardioSTAT (Icentia, Quebec, QC) device at 8:00, 12:00, and 20:00 daily. Mean ± SEM. *P* = 0.40. OZA, standard-of-care + ozanimod; SOC, standard-of-care alone.

No difference occurred in the median PR duration (140 msec [IQR 132-155] vs 146 msec [IQR 131-165] in the SOC vs the OZA group, respectively; *P* = 0.27), nor in the median QT duration (442 msec [IQR 435-451] vs 434 msec [IQR 419-454], respectively; *P* = 0.18) between the 2 groups during the first-10-days monitoring period (Table 2; Figure 2, Figure 3). No significant increase occurred in QT duration during monitoring (Fig. 3). The median QT duration at day 3 was not statistically different between the 2 groups (442 msec [IQR 433-451] in the SOC group vs 441 msec [IQR 423-457] in the OZA group; *P* = 0.45), nor was the PR duration at day 3 (141 msec [127-161] vs 145 msec [IQR 131-165]; *P* = 0.68; Figure 2, Figure 3; Table 2).

One patient in the OZA group, and 2 in the SOC group, experienced AF during cardiac recording; none of them was known to have a history of AF. Ozanimod does not appear to enhance the occurrence of AF events. No significant ventricular arrhythmias or symptomatic rhythmic events were reported.

### Clinical course and hemodynamic impact

The detailed outcome data of the main COZI study are reported in another publication.^9^ Respiratory deterioration leading to intubation occurred in 1 patient (5%) in the OZA group vs in 5 patients (22%) in the SOC group (*P* = 0.10). The intubated patient in the OZA group was excluded from the analysis because his CardioSTAT was lost. No patient in OZA group needed hemodynamic support, vs 6 (26%) who did need this support in the SOC group. Hemodynamic support was mainly needed during intubation. During hospitalization, arterial pressures were significantly lower in the SOC group vs those in the OZA group (Table 2).

Four transient episodes of hypotension, with mean arterial pressure (MAP) < 65 mm Hg, were observed in the OZA group, compared to 6 episodes in the SOC group. No bradycardia < 55 bpm was associated with hypotensive events.

### Cardiac adverse events

Two cardiac adverse events occurred in the OZA group, vs none in the SOC group. The first patient had asymptomatic type 1 second-degree AVB overnight at day 7. The maximum pause recorded during this episode was 2.4 seconds. The patient was a 66-year-old man with no cardiac history. Bradycardia occurred 18 hours after the ozanimod dose was increased. The dose was reduced the next day, and no recurrence of AVB was noted afterward. No electrolyte abnormality was recorded.

The second patient had an asymptomatic sinus pause of 3.35 seconds, which occurred overnight at day 3. The patient was an 80-year-old man with a history of hypertension. The ozanimod dose was increased, following protocol, on day 5, without recurrence of significant pause.

Neither patient took beta-blockers, nondihydropyridine calcium-channel blockers, or antiarrhythmics drugs. No hemodynamic instability and no clinical consequence of these episodes were observed. Ozanimod was administered mainly in the morning (88% of drug administrations). No association was found between the time of day of ozanimod administration and the bradycardia observed.

## Discussion

The COZI study is the first randomized trial to initiate an SRL treatment in patients with an active viral infection. The key findings of our substudy of the COZI trial are that, in patients with hypoxemia related to SARS-CoV-2 viral pneumonia requiring oxygen, ozanimod use is not associated with significant conductive disorders, is not associated with QT interval prolongation, and is associated with slight nocturnal bradycardia without clinical consequences.

In this study, we found that the OZA group had a significantly lower mean hourly HR, and the length of time this group spent at < 60 bpm was greater than that in the SOC group.

The OZA group had a maximal HR decrease 3 days after treatment initiation. On prolonged administration, the HR stabilized. This decrease in HR was not observed on the days following the dose increase, but the HR difference between the SOC group and the OZA group was maintained until study day 10 (the last day analyzed).

These findings are consistent with those of the phase 1 trial assessing ozanimod’s effect on healthy subjects^8^ and randomized clinical trials.^11^, ^12^, ^13^ No significant (< 30 bpm) or clinically meaningful bradycardia was observed. Some studies with fingolimod suggested that this negative chronotropic effect may attenuate over time, secondary to S1P desensitization on atrial myocytes—the “first dose effect.”^8^^,^^14^ Some authors suggested that fingolimod may cause functional antagonism with chronic drug exposure, due to receptor internalization, that would decrease cardiac side effects given the duration of the treatment.^15^

To our knowledge, no experimental studies observed this phenomenon with ozanimod use, and its impact on shingosine-1-phosphate receptor ligand may differ in the context of acute infectious disease. This observation requires further investigation. Bradycardia attenuation after a few days of treatment seems to be confirmed in randomized trials,^11^, ^12^, ^13^ and a gradual dose escalation of ozanimod may minimize initial bradycardia. A phase 1 randomized, double-blind, placebo-controlled study in healthy adult subjects showed no QTc interval prolongation and no clinically significant bradycardia.^16^ In our study, we showed that no difference occurred between the median of the mean QT interval and PR interval duration during cardiac monitoring. Therefore, our analyses support the concept that the cardiac innocuity of ozanimod use that has been seen previously in healthy subjects, in multiple sclerosis and in ulcerative colitis, likely transfers to the current hospitalized COVID-19 population.

We observed a maximum reduction in HR at night, respecting the physiological nychthemeral variations in cardiac rhythm, which supports the inconsequential nature of ozanimod-induced bradycardia. Circadian periodicity respect supports a good level of functioning of the autonomic nervous system. Indeed, in sinus node dysfunction, an alteration occurs in the nychthemeral cycle proportional to the dysfunction.^17^

No association between the time of day of drug administration and significant bradycardia was found, but only a small sample of patients received ozanimod in the afternoon, resulting in a peak plasma concentration overnight. Assuming maximum bradycardia occurs at night, administration of the treatment in the morning would be appropriate.

### Future directions

In sepsis, induced tachycardia impairs ventricular filling and increases myocardial oxygen requirements. A recent meta-analysis showed that ultrafast-acting β1-blockers reduced mortality in septic patients who have persistent tachycardia.^18^ Ivabradine also has been studied in this indication, because it preserves blood pressure, cardiac output, and left ventricular systolic function.^19^ Ozanimod-induced bradycardia in patients with SARS-CoV-2 viral or other type of pneumonia or sepsis may have a beneficial effect on their management.

### Limitations

Clinical courses, such as respiratory failure and hemodynamic instability, are confounding factors in HR analysis. The main limitations are related to the small size of our sample. Lack of power in our analyses may cause bias in the results. The limited cohort size also made multivariate analysis irrelevant because of the lack of events. Moreover, the only patient with an initially unfavourable evolution requiring intubation in the OZA group was excluded from the analysis because his CardioSTAT was lost, creating a potential bias in the results. Furthermore, due to the highly selective inclusion and exclusion criteria of patients, in particular, the inclusion of patients without heart disease, the external validity of this study is limited.

## Conclusion

Ozanimod use in COVID-19 patients showed a maximal reduction in HR 3 days after the first ozanimod administration, without clinically relevant cardiac adverse events. Larger trials are needed to confirm these results.
